# Vestibular Modulation of Long-Term Potentiation and NMDA Receptor Expression in the Hippocampus

**DOI:** 10.3389/fnmol.2020.00140

**Published:** 2020-08-11

**Authors:** Paul F. Smith, Bruno Truchet, Franck A. Chaillan, Yiwen Zheng, Stephane Besnard

**Affiliations:** ^1^Department of Pharmacology and Toxicology, School of Biomedical Sciences, Brain Health Research Centre, University of Otago, Dunedin, New Zealand; ^2^Brain Research New Zealand, The Eisdell Moore Centre for Hearing and Balance Research, University of Auckland, Auckland, >New Zealand; ^3^Aix Marseille University, CNRS, LNC UMR 7291, FR 3C FR 3512, Marseille, France; ^4^Université de Normandie, INSERM U 1075 COMETE, Caen, France

**Keywords:** hippocampal long-term potentiation, NMDA receptors, bilateral vestibular loss, dentate gyrus, CA1, E-S potentiation

## Abstract

Loss of vestibular function is known to cause spatial memory deficits and hippocampal dysfunction, in terms of impaired place cell firing and abnormal theta rhythm. Based on these results, it has been of interest to determine whether vestibular loss also affects the development and maintenance of long-term potentiation (LTP) in the hippocampus. This article summarizes and critically reviews the studies of hippocampal LTP following a vestibular loss and its relationship to NMDA receptor expression, that have been published to date. Although the available *in vitro* studies indicate that unilateral vestibular loss (UVL) results in reduced hippocampal field potentials in CA1 and the dentate gyrus (DG), the *in vivo* studies involving bilateral vestibular loss (BVL) do not. This may be due to the differences between UVL and BVL or it could be a result of *in vitro/in vivo* differences. One *in vitro* study reported a decrease in LTP in hippocampal slices following UVL; however, the two available *in vivo* studies have reported different results: either no effect or an increase in EPSP/Population Spike (ES) potentiation. This discrepancy may be due to the different high-frequency stimulation (HFS) paradigms used to induce LTP. The increased ES potentiation following BVL may be related to an increase in synaptic NMDA receptors, possibly increasing the flow of vestibular input coming into CA1, with a loss of selectivity. This might cause increased excitability and synaptic noise, which might lead to a degradation of spatial learning and memory.

## Introduction

Numerous studies over the last two decades have shown that damage to the peripheral vestibular system, especially bilateral lesions, results in spatial memory impairment in both animals and humans (for reviews see Besnard et al., 2016; Smith, 2017; Agrawal et al., 2020). This memory impairment has been attributed to the effects of vestibular loss on the hippocampus, although many other areas of the medial temporal lobe and cortex are likely to be involved as well. Many different studies have provided evidence that vestibular information reaches the hippocampus *via* multiple pathways that include the thalamus, theta rhythm-related structures and probably the cerebellum (Cuthbert et al., 2000; Rancz et al., 2015; Leong et al., 2019; for a review see Hitier et al., 2014). In the early 2000s it was reported that bilateral vestibular loss (BVL) in rats resulted in a substantial dysfunction of hippocampal place cells (Stackman et al., 2002; Russell et al., 2003) as well as theta rhythm (Russell et al., 2006; Neo et al., 2012; Tai et al., 2012). Theta rhythm has been reported to be abnormal in the entorhinal cortex, suggesting the possibility that grid cells are also dysfunctional following BVL (Jacob et al., 2014). Head direction cells in the thalamus also function abnormally following vestibular loss (for a review see Cullen and Taube, 2017). A number of studies in humans have shown that vestibular disorders are associated with various forms of hippocampal atrophy, depending on the specific condition (for a review see Smith, 2017). Therefore, there is ample reason to think that the loss of vestibular function impairs normal hippocampal function and its role in spatial memory.

In addition to the effects of BVL on hippocampal place cells and theta rhythm, an obvious question is whether it has any effect on long-term potentiation (LTP), given the accepted role of LTP in spatial memory (for reviews see Bliss and Collingridge, 1993; Lynch, 2004; Nicoll, 2017). Despite the demonstrations that BVL causes place cell dysfunction, published in 2002 and 2003 (Stackman et al., 2002; Russell et al., 2003), only three studies have addressed this question directly over the last two decades, and their results are inconsistent. The objective of this review is to summarize, compare and critically evaluate these three studies, and other related evidence, to develop a more cohesive view of the role of the vestibular system in the modulation of hippocampal LTP.

## Early Studies of The Effects of Vestibular Lesions on Hippocampal Field Potentials

Zheng et al. (2003) published a study in which they had performed unilateral surgical vestibular lesions (UVLs) in rats and then removed hippocampal slices at 4–6 weeks or 5–6 months following the lesion. The rationale of the study was to investigate the acute and longer-term effects of UVL on neuronal excitability in the hippocampus *in vitro*. They recorded field potentials in CA1 in response to electrical stimulation of the Schaffer collaterals and analyzed both the field excitatory postsynaptic potentials (fEPSPs) and the population spikes (PSs), the former reflecting the efficacy of the dendritic synaptic inputs and the somal field EPSP (sfEPSP) and the latter, the somal effects of the stimulation.

The input/output (I/O) curves from slices taken from UVL rats exhibited a significant reduction in the PS spike amplitude compared to naïve animals and those that had undergone sham surgery. This was the case for both time points and by 5–6 months, it occurred on the side contralateral to the lesion as well as ipsilaterally. The results were similar for the sfEPSP slopes at 5–6 months.

Ipsilaterally, there was a significant increase in paired-pulse inhibition in the 5–6 month UVL group at the shortest inter-stimulus interval (ISI); however, at longer ISIs there were significant increases in paired-pulse facilitation compared to age-matched controls and the 4–6 week group. On the contralateral side, there was increased paired-pulse inhibition for the shortest ISIs in the 5–6 month group, as well as increased paired-pulse facilitation for all ISIs.

This was the first study to report dramatic changes in electrical excitability in the hippocampus, following UVL, in hippocampal slices *in vitro*. The fact that the decrease in PS amplitude and fEPSP and sfEPSP slopes was bilateral was surprising and suggested that both sides of the hippocampus, or at least CA1, were affected by a UVL and that therefore it receives input from each vestibular labyrinth *via* the vestibular nucleus and/or cerebellum. Furthermore, the effects appeared to become greater at the longer time point. This study included both sham surgery controls and also naïve controls, and therefore involved a robust design. The paired-pulse analysis reflects recurrent and feedforward inhibition as well as changes in glutamate release underlying facilitation. The paired-pulse results obtained in this study suggested that UVL may cause changes in presynaptic neurotransmitter release. Because the study was correlational, it is difficult to determine whether any of these changes represented simply the effects of UVL or some form of compensation for it.

## Effects of Vestibular Stimulation on Hippocampal LTP

Based on an earlier study by Horii et al. (1994), which showed that electrical stimulation of the round window in rats could evoke acetylcholine (ACh) release in the hippocampus, Tai and Leung (2012) investigated whether natural vestibular stimulation, in the form of whole-body rotation, could modulate LTP in CA1. The rationale underlying the study was to investigate the role of cholinergic input in hippocampal LTP and whether activation of ACh receptors would increase its magnitude. LTP induction was performed in freely behaving rats who received vestibular stimulation and the results were compared to alert rats who were immobile. They observed that the LTP induced during natural vestibular stimulation was greater than that induced in immobile animals and that pretreatment with atropine, a muscarinic acetylcholine (mACh) receptor antagonist, eliminated this effect, as did selective lesions of the cholinergic neurons in the medial septum. These results suggested that the enhancement of LTP caused by natural vestibular stimulation was mediated by cholinergic input to the hippocampus from the medial septum. So far, this is the only study to investigate the effects of vestibular stimulation on hippocampal LTP.

## Effects of Vestibular Lesions on Hippocampal LTP

In the first systematic study of the effects of complete vestibular loss (BVL) on hippocampal LTP, Zheng et al. (2010) used both anesthetized and alert rats and investigated LTP in both CA1 and the dentate gyrus (DG). The rationale for this study was to investigate whether complete loss of peripheral vestibular input would impact negatively on LTP. They studied LTP in freely moving rats up to 43 days post-BVL and anesthetized rats at 7 months post-BVL. It is important to note that the BVL involved surgical lesions (see below).

**Figure 1:**
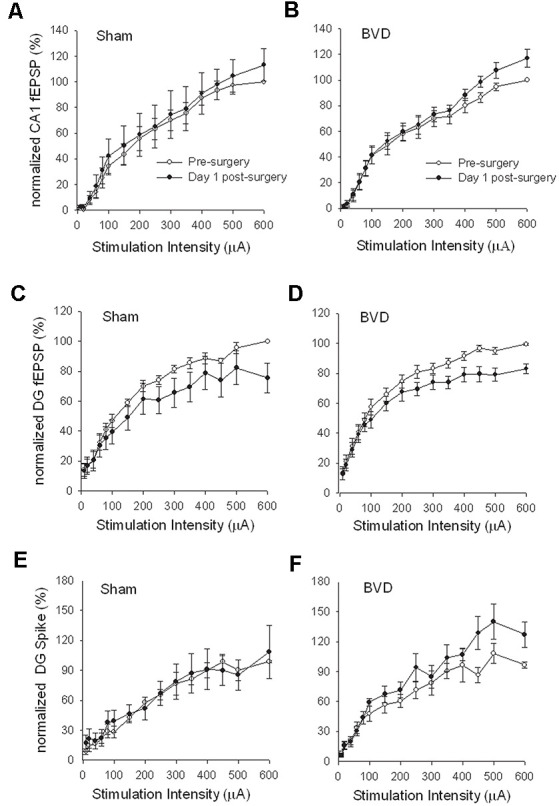
Input/output (I/O) curves for sham **(A,C,E)** and bilateral vestibular deafferentation(BVD; **B,D,F**) animals before (open circles) and 1 day after (filled circles) BVD or sham surgery. I/O curves collected in freely moving animals are presented for fEPSP recordings in CA1 **(A,B)** and dentate gyrus (DG; **C,D**) and population spike recordings in the DG **(E,F)**. Each response has been normalized to the response obtained at the highest stimulus intensity (600 μA) in the presurgery I/O curve. ANOVA revealed no effects of BVD surgery on any of these measures. Data represent mean ± SEM, for this and all subsequent figures. *n* = 5–6 for sham group and *n* = 5–9 for BVD group. From Zheng et al. (2010) with permission.

**Figure 2:**
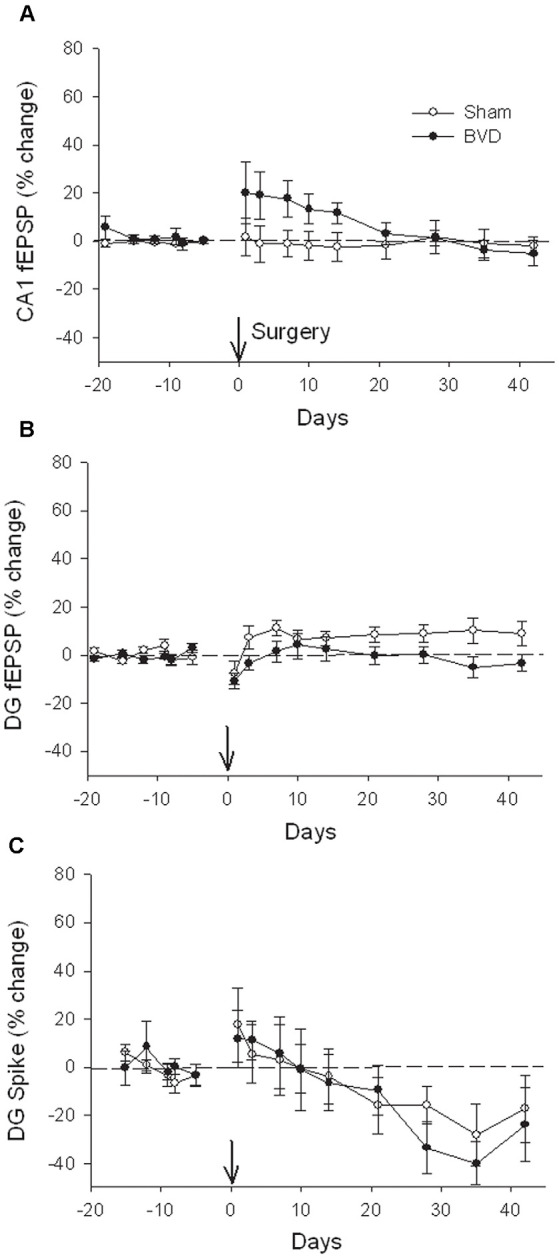
Postsurgical stability of field potential recordings in freely moving animals. Field EPSPs (CA1 and DG, **A** and **B**, respectively) and population spikes (DG, **C**) were recorded in sham (open circles) and bilateral vestibular deafferentation (BVD; filled circles) animals for 40 days following surgery (arrow). ANOVA revealed no significant differences between groups across time for any measure. In both groups, the population spikes gradually declined on average over this period. *n* = 5–6 for sham group and *n* = 5–8 for BVD group. From Zheng et al. (2010) with permission.

**Figure 3:**
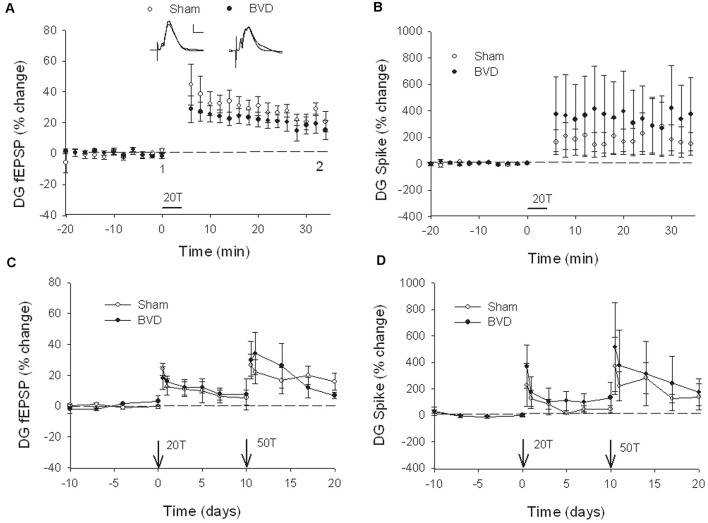
Induction and persistence of perforant path long-term potentiation (LTP) in freely moving animals. Tetanisation with 20 trains of high-frequency stimulation (HFS; 20T) produced equivalent initial potentiation across groups for both the field excitatory postsynaptic potential (fEPSP; **A**) and population spike measure **(B)**. There was also no difference between groups in the decay of the LTP over 10 days **(C,D)**. Subsequent tetanisation with a stronger protocol (50T) produced greater LTP, but this was again of equivalent induction and persistence across groups **(C,D)**. Inset representative waveforms are averages of 15 sweeps taken from individual animals in each group, either just before (1) or 30 min (2) after HFS. Calibration bars: 5 μs, 5 mV. *n* = 5 for sham group and *n* = 5–6 for BVD group. From Zheng et al. (2010) with permission.

In terms of the I/O curves for the chronic, alert animals, there were no significant differences for the normal fEPSP slopes or PS amplitudes in either CA1 or the DG following BVL, across the 43 day recording period (Figures 1, 2). When LTP was induced in the DG using 20 trains of high-frequency stimulation (HFS) in the perforant path (400 Hz, 25 ms, pulse duration 250 μs), there was no significant difference in either the size or the decay of the LTP (Figure 3).

**Figure 4:**
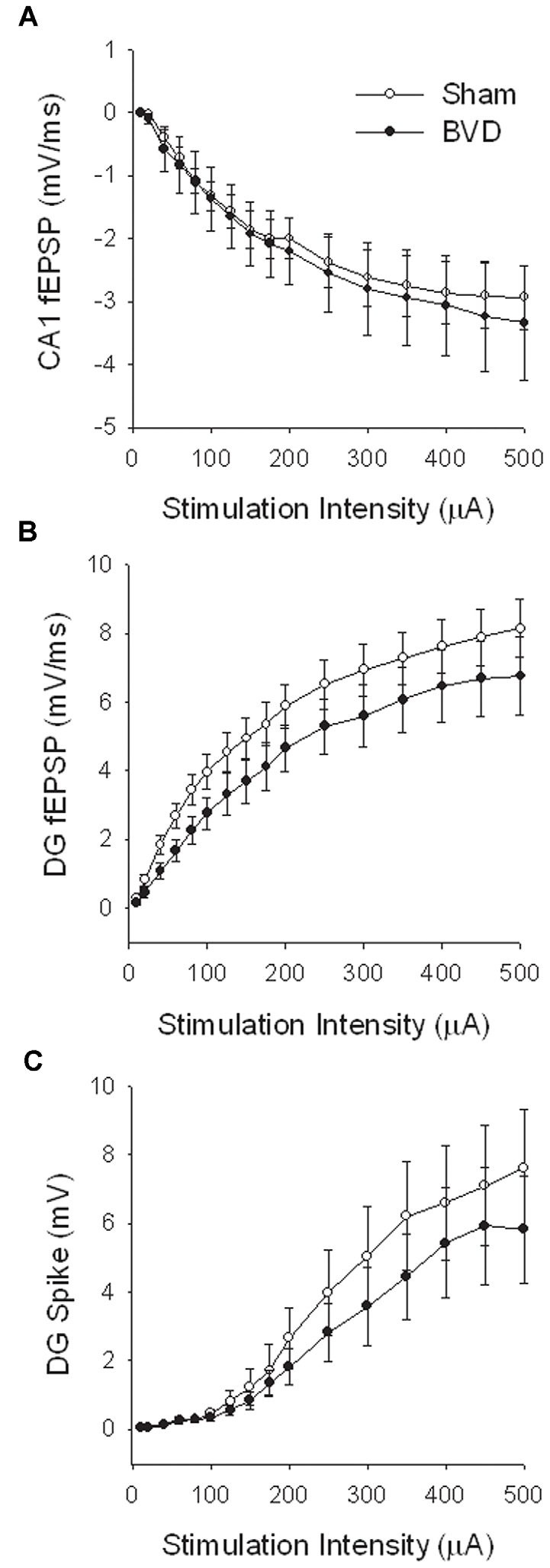
Input-output analysis in anesthetized animals, 7 months after surgery. There were no significant differences in fEPSP slope for either CA1 **(A)** or DG **(B)**, or in DG population spike height **(C)**, between the sham (open circles) and BVD (filled circles) groups. *n* = 5–7 for sham and BVD groups, respectively. From Zheng et al. (2010) with permission.

**Figure 5:**
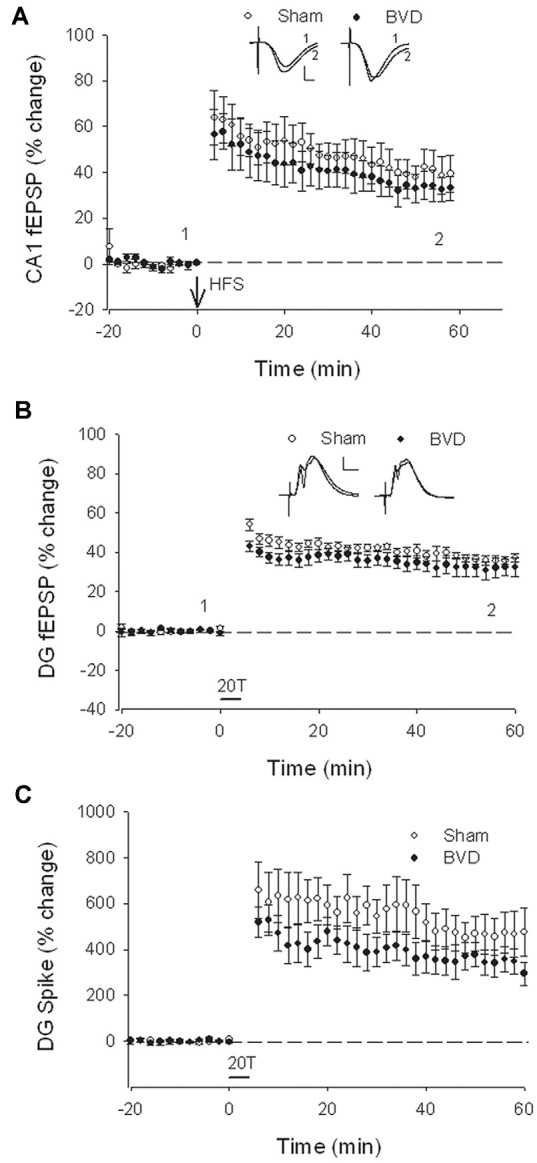
Induction of LTP in anesthetized animals, 7 months after surgery. HFS (100 Hz) in neither the Schaffer collaterals **(A)** nor the perforant path (20T, **B,C**) produced differential LTP between sham (open circles) and BVD (closed circles) animals. Inset representative waveforms are averages of 15 sweeps taken from single animals in each group, either just before (1) or 60 min (2) after HFS. Calibration bars: CA1, 4 μs, 4 mV; DG, 5 μs, 5 mV. *n* = 5–7 for sham and BVD groups, respectively. From Zheng et al. (2010) with permission.

At 7 months post-BVL in acute, anesthetized animals, there was again no significant difference in the fEPSP slope or the PS amplitude in the DG or CA1 between BVL and sham animals (Figure 4). Following the induction of LTP, although the slope of the fEPSP in CA1 and the amplitude of the PS in the DG appeared smaller than for sham animals, the differences were not statistically significant (Figure 5).

These results were somewhat surprising, especially in light of the earlier study by Zheng et al. (2003), where clear decreases in the normal fEPSP slope and PS amplitude were observed in hippocampal slices from UVL rats. However, UVL and BVL are quite different forms of vestibular lesions. In the case of UVL, one peripheral vestibular system is eliminated, creating a dramatic imbalance in activity between the vestibular nuclei (VN) on the ipsilateral and contralateral sides. This generates a behavioral syndrome that includes spontaneous ocular nystagmus and a postural asymmetry directed toward the lesioned side. Amongst other effects, the experience of this syndrome is stressful. Following BVL, although vestibular reflexes such as the vestibulo-ocular reflexes (VORs) are abolished, there is no asymmetry in neuronal activity between the bilateral VNs. Thus, the impact of UVL and BVL on the hippocampus could be quite different. A second difference of course is that the study involving UVL was *in vitro*, whereas the BVL study was *in vivo*. *In vitro* studies of the hippocampus necessarily mean that all remaining vestibular input, as well as that from all other cranial nerves, is removed, so that if the vestibular lesion was a UVL, then the vestibular input from the other ear is removed as well.

Lee et al. (2017) conducted an *in vitro* study of the effects of surgical UVL in rats on LTP in CA1 in hippocampal slices. The rationale for this study was to investigate whether unilateral loss of vestibular input would affect LTP induced in the hippocampus, *in vitro*. They used three time-points: 1 day, 1 week, and 1 month following UVL and a microelectrode array was used to record field potentials. Using I/O curves, they found that bilateral slices from animals at 1 day and 1-week post-UVL exhibited reductions in the fEPSP amplitudes compared to sham controls, although the decreases were more obvious on the ipsilateral side at 1-day post-UVL. Theta-burst stimuli (TBS) were used to induce LTP. These consisted of three pulse trains at 20-s intervals with 10 bursts applied at 5 Hz per train and four pulses applied at 100 Hz per burst. In slices from UVL animals, the induction rate of LTP was only 56.9 ± 8.6%, compared to 85.3–88.8% in slices from sham animals. The reduction in the rate of LTP induction occurred at 1-week post-UVL and was present bilaterally.

The pre-LTP field potential I/O results from this study are consistent with those obtained *in vitro* by Zheng et al. (2003). In both cases, a UVL was performed and then hippocampal slices removed. However, the LTP results of Lee et al. (2017) appear inconsistent with those obtained by Zheng et al. (2010) *in vivo*. It is difficult to compare the two studies given the difference in the vestibular lesions used (i.e., UVL vs. BVL, see “Discussion” above), the *in vitro/in vivo* difference (see “Discussion” above), but also the nature of the stimuli used for LTP induction was completely different. Zheng et al. (2010) used HFS stimulation (400 Hz, 25 ms, pulse duration 250 μs) of either the perforant path or the Schaffer collaterals, whereas Lee et al. (2017) used TBS consisting of three pulse trains at 20-s intervals with 10 bursts applied at 5 Hz per train and four pulses applied at 100 Hz per burst, in the Schaffer collaterals only. The methods of recording were quite different. Although Zheng et al. (2003) used a conventional recording electrode and Lee et al. (2017) employed a multi-electrode array (a MED64 microelectrode array, 8 × 8), the electrodes had the same features (50 μm diameter Teflon-coated); hence this parameter could not alter the recorded signals. However, biphasic pulses were not delivered at the same frequency (0.17 Hz, Zheng et al., 2003; 0.05 Hz, Lee et al., 2017). A short interval between two pulses can affect the evoked potential (as during a paired-pulse procedure, range 0–1,000 ms) but not with the range used (6 s or 20 s). Nevertheless, three points could partially explain the discrepancies. First, recordings were performed with a 21–23°C ACSF by Zheng et al. (2003) and 30°C by Lee et al. (2017), which can affect the membrane properties of the neurons and thus the evoked potential (Deisz, 1999; Volgushev et al., 2000; Liebregts et al., 2002). Second, the time points were different, with Zheng et al. (2003) using 4–6 weeks or 5–6 months following the lesion and Lee et al. (2017) using 1 day, 1 week, and 1 month (equivalent to 4–6 weeks) post-lesion. At such time-points, differences in post-lesion edema and/or gliosis processes could develop (Steward et al., 1986; Brace et al., 1997; Tourdias et al., 2011). Finally, the rat strains were different (Wistar vs. Sprague–Dawley), which could have led to electrophysiological discrepancies (Guitart et al., 1993; Potier et al., 1993; Fuzik et al., 2013; Bruzos-Cidón et al., 2014). Despite the methodological differences, the two studies showed similar changes in excitability in the hippocampus, albeit that LTP induction was not specifically measured in Zheng et al. (2003).

**Figure 6:**
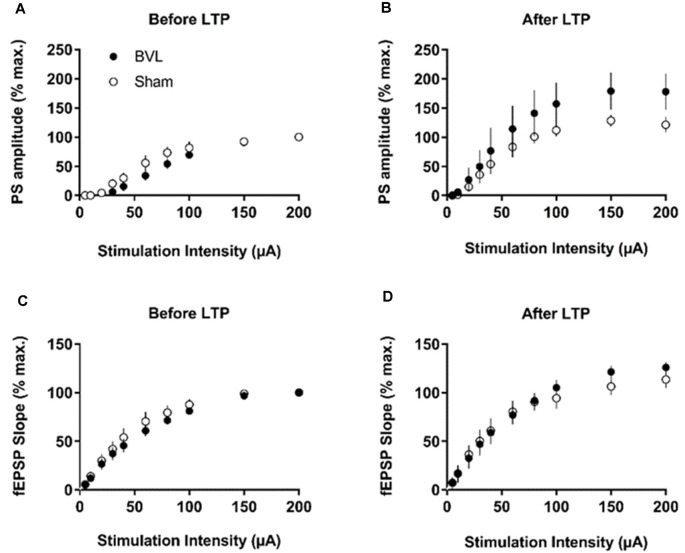
IO curves for the population spike (PS) amplitude **(A,B)** and fEPSP slope **(C,D)**, before and after the entire LTP procedure. The IO obtained by a single pulse across an intensity range of 5–200 μA. Measures were repeated four times, every 15 s, normalized for each rat concerning the maximum value (obtained for a 200 μA stimulation intensity), and averaged for each stimulation intensity. Data are expressed as mean ± SEM (BVL: *n* = 7; Sham, *n* = 7). From Truchet et al. (2019) with permission.

**Figure 7:**
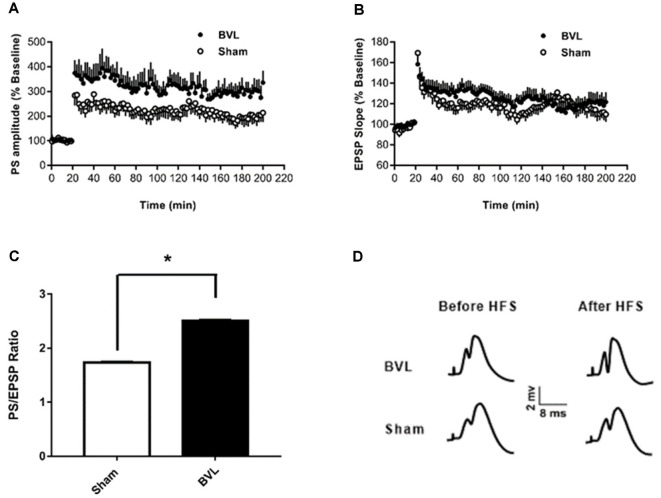
LTP. **(A,B)** DG recording obtained before (10 min baseline) and after (3 h) weak tetanus (arrow) of the medial performant path (MPP). Data are expressed as mean ± SEM (BVL: *n* = 7; Sham, *n* = 7). For clarity, each point represents the mean of four plotted measures. Data were normalized concerning the baseline level. **(A)** Percentage changes of the population spike (PS) amplitude. **(B)** Percentage changes of the fEPSP slope. **(C)** E-S potentiation. The (Population spike amplitude)/(EPSP Slope) ratio was calculated using normalized values, expressed in percentage of the baseline for each animal, for each stimulation. Bar graph of the mean ES ratio obtained from the last (third) hour of post-HFS recording (BVL: *n* = 7; Sham, *n* = 7). Error bars represent SEM (*P = 0.013). **(D)** Representative traces of potential evoked in the DG by stimulation of the MPP, before (baseline) and after HFS (LTP induction), in both groups. From Truchet et al. (2019) with permission.

In the most recent study, Truchet et al. (2019) investigated the effects of BVL on LTP in anesthetized rats at 30 days following BVL. The rationale for this study was to investigate the effects of complete loss of vestibular function on LTP *in vivo*, at a time when some degree of vestibular compensation had developed. They employed a chemical lesion procedure in which sodium arsanilate is injected through the tympanic membrane. They measured the fEPSP slopes and PS amplitudes in the DG before and after LTP induction. A critical difference from the study by Zheng et al. (2010) was that they used a “moderate” HFS protocol for LTP induction. This consisted of two main trains at a 1 min intertrain interval, each main train consisting of five subtrains at a 1 Hz inter-subtrain interval. Each subtrain contained a burst of 10 biphasic (+250 μs/−250 μs) pulses at 400 Hz. The logical consequence was a lower level of LTP (around 20% vs. 40% fEPSP increase, around 120% vs. 600% PS amplitude increase). This was designed to avoid “LTP saturation” so that increases and decreases in LTP could be detected if they were produced. LTP was measured throughout 3 h post-induction, with the first 5 min excluded due to the possibility of post-tetanic potentiation (PTP; McNaughton, 1982). They observed no significant differences between the BVL and sham groups for any of the stimulation intensities used for the I/O curves in the DG, either before or following the induction of LTP. This was the case for both the fEPSP slope and the PS amplitude (see Figure 6). There were few differences in the paired-pulse ratios between the BVL and sham rats. Following LTP, the mean fEPSP slope was increased over the 3 h of recording but this was equally so for both BVL and sham animals (Figure 7). By contrast, the level of PS potentiation was higher for the BVL group, and this became more prominent with time (Figure 7). EPSP-Spike (ES) Potentiation is a phenomenon in which the ratio of the PS amplitude to the fEPSP slope, increases. After the induction of LTP, there was a tendency for a higher E-S potentiation in the BVL group compared to the sham group, which was more prominent during the final hour of recording (Figure 7).

## The Possible Role of N-Methyl-D-Aspartate (NMDA) Receptors in Hippocampal LTP Changes Following UVL or BVL

An early western blotting study demonstrated that the expression of the GluN 1 and GluN 2A subunits of the N-methyl-D-aspartate (NMDA) subtype of glutamate receptor, decreased in the ipsilateral CA2/3 region at 2 weeks following surgical UVL, while the expression of the GluN 2A subunit was also reduced in the contralateral CA2/3 region (Liu et al., 2003). On the other hand, the expression of the GluN 2A subunit was increased in the CA1 region at 10 h following UVL (Liu et al., 2003). The rationale for these early studies was to explore potential changes in hippocampal NMDA receptors following a UVL, given that these receptors were already known to be important in LTP.

With a similar rationale in mind, Besnard et al. (2012) measured NMDA receptor density and affinity using receptor autoradiography at 2 months following BVL. They employed a sequential chemical UVL procedure, involving intratympanic sodium arsanilate injections (i.e., one ear, followed several weeks later by the other ear), and following the second UVL, resulting in a BVL, they observed a significant increase in the NMDA receptor B_max_ and a decrease in K_d_ in the hippocampus. In a study using simultaneous surgical BVL, Zheng et al. (2013) used western blotting and could find no significant differences in the expression of the GluN 1, GluN 2A, GluN 2B, GluA 2, GluA 3 or GluA 4 subunits in the CA1, CA2/3 or DG subregions of the hippocampus at 24 h, 72 h, 1 week, 1 month or 6 months following BVL.

**Figure 8:**
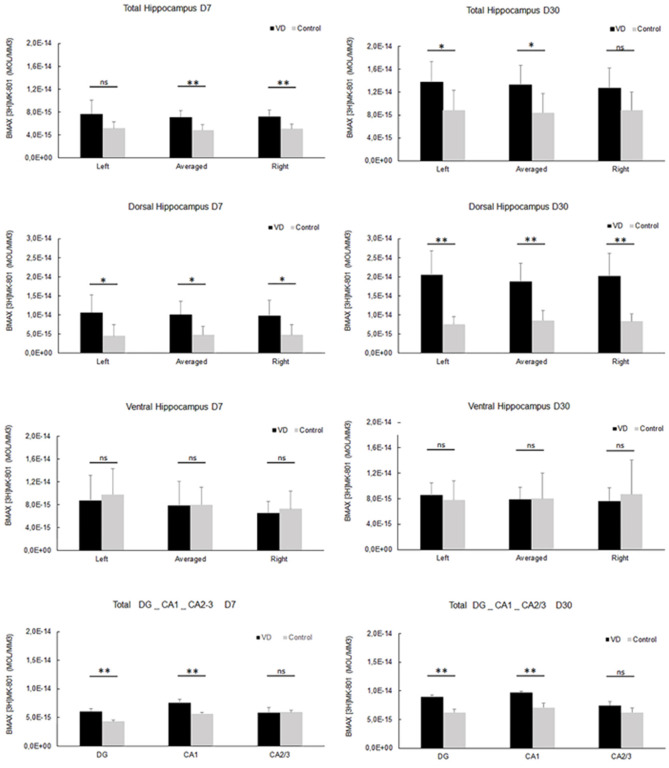
NMDA receptor quantitative autoradiography. Time course of hippocampal NMDA receptor density expressed in mol/mm^3^ at 7 days (left panel) and 30 days (right panel) following trans-tympanic bilateral injection of arsanilate (vestibular deficiency/VD in black *n* = 8) or of saline solution (control in grey, *n* = 7). NMDA receptor density was calculated from the total hippocampus, the left, and right side separately, and the dorsal and ventral parts, the DG, the CA1 and CA2/3 sublayers (combining the right and left parts of the sublayers, due to the lower surface of beta-emission). Statistical abbreviations: ns, non-significant, *p < 0.5, **p < 0.02. Error bars represent SD. From Truchet et al. (2019) with permission.

**Figure 9:**
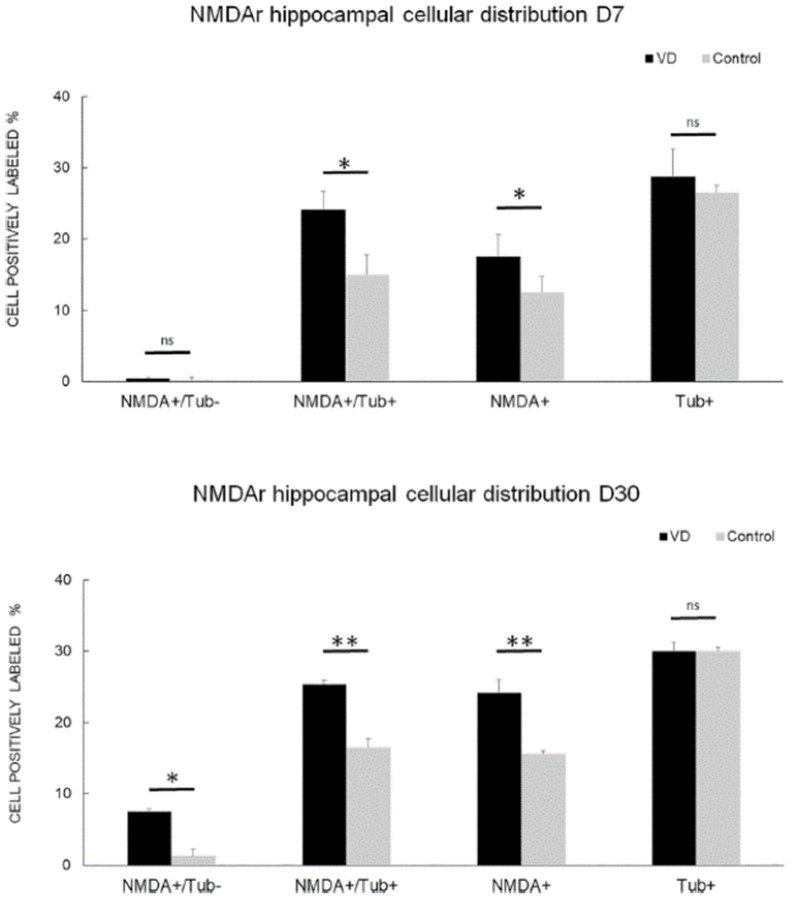
NMDA receptor neuronal distribution by flow cytometry. Time course of the distribution of NMDA receptors (NMDA+) on neurons (Tub+) and non- neuronal cells (Tub-) at 7 days (top panel) and 30 days (bottom panel) following trans-tympanic bilateral injection of arsanilate (vestibular deficiency/VD in black = 8) or of saline solution (control in gray, *n* = 7). Results are expressed in percentage of cell samples for each group (VD and control) and labeled for NMDA+/Tub− (non-neuronal cells expressing NMDA receptors), NMDA+/Tub+ (neurons expressing NMDA receptors), NMDA+ (% of cells expressing NMDA receptors irrespective of the type of cells), Tub+ (% of neurons among the cells’ samples analyzed). Tub+ = cells positive for beta-Tubulin, a neurofilament marker specific the neurons. Statistical abbreviations: ns, non-significant, *p < 0.5, **p < 0.02. Error bars represent SD. From Truchet et al. (2019) with permission.

In the most recent study by Truchet et al. (2019), NMDA receptor expression was investigated in the hippocampus at 7 and 30 days following BVL, using receptor autoradiography as well as flow cytometry. The rationale for these experiments was to determine whether NMDA receptor expression might change differentially at short and longer-term time points and also to correlate NMDA receptor expression with the LTP observed at 30 days post-BVL. These experiments were done as part of the same study that measured changes in LTP following BVL (Truchet et al., 2019); however, separate animals were used for the LTP experiments. The NMDA receptor B_max_ was significantly increased in the dorsal hippocampus at D_7_ and D_30_ but was higher in the left than the right dorsal hippocampus at D_7_ and D_30_ compared to controls (Figure 8). This increase was also seen when the whole right and left hippocampi were analyzed separately, except that the NMDA receptor upregulation did not reach statistical significance in the left hippocampus at D_7_ or in the right one at D_30_. This might have been due to the relative lack of change in the ventral hippocampus. A significant increase in NMDA receptor density was observed in CA1 at D_7_ and D_30_ and also in the DG; however, it remained unchanged in the CA2/CA3 at D_7_ and D_30_. From the flow cytometry studies, the number of neurons expressing NMDA receptors was selectively increased in the hippocampus at D_7_ and D_30_ (see Figure 9). Similar results were reported by Benoit et al. (2018a).

None of these studies was directly related to measurements of hippocampal LTP. Therefore, it is difficult to interpret their functional significance. Truchet et al. (2019) suggested that the increase in NMDA receptors may underlie the ES potentiation observed in the hippocampus. If NMDA receptors were up-regulated synaptically, then there would be increased excitation of DG neurons that might lead to a greater PS for a given amount of excitation reflected in the perforant path fEPSP, i.e., the size of the PS would not specifically reflect the stimulated input, which would generate increased synaptic noise.

Once again, the differences between UVL and BVL used in the different studies are important, and the sequential UVLs used by Besnard et al. (2012) are difficult to compare to the simultaneous BVL used by Zheng et al. (2013). There are also substantial differences between surgical and chemical lesions of the peripheral vestibular system (see below).

It is worth noting that BVL has also been demonstrated to result in the down-regulation of M_1_ mACh receptors across all subregions of the hippocampus (Aitken et al., 2016). This is consistent with the results of Tai and Leung (2012), who reported that a mACh antagonist blocked the enhancement of LTP induced by natural vestibular stimulation and that lesions that eliminated the cholinergic input from the medial septum, also had this effect. Aitken et al. (2017) also reported that although there was no significant change in ACh release into the hippocampus following BVL, there was a surprising increase in the number of cholinergic neurons in the pedunculopontine tegmental nucleus, an important part of the cholinergic pathway that generates theta activity. The functional significance of this finding is unknown.

## Discussion

The investigation of the nature of LTP in the hippocampus following UVL or BVL was motivated by studies showing spatial memory deficits (for reviews see Besnard et al., 2016; Smith, 2017; Agrawal et al., 2020) and hippocampal place cell dysfunction (Stackman et al., 2002; Russell et al., 2003) in animals with vestibular loss. It made sense intuitively that if spatial memory processing in the hippocampus was disrupted, then the mechanisms underlying LTP might be disrupted as well. To date, the few studies conducted have yielded far more complex results. The studies are difficult to compare directly because of the different uses of UVL vs. BVL, *in vitro* vs. *in vivo* models of hippocampal function, different time points post-lesion, and different methods of inducing vestibular dysfunction (chemical vs. surgical labyrinthectomy). Therefore, the available studies can only be compared to those other studies using similar methods, if not the same.

### *In vitro* Studies

In terms of the two *in vitro* studies, what they have in common is that they removed hippocampal slices from rats that had received a UVL, at similar time points, and they recorded field potentials in the slices at various times post-lesion. Both Zheng et al. (2003) and Lee et al. (2017) reported that the fEPSP slope was reduced following UVL, suggesting a decrease in excitability, *in vitro*, as a result of the lost vestibular input. In both cases, the effect was bilateral, which supports the evidence that vestibular information from one vestibular labyrinth is transmitted to both hippocampi (Cuthbert et al., 2000; Rancz et al., 2015). It is noteworthy that there are several differences between the two studies. The methods of recording were quite different, with Zheng et al. (2003) using a conventional recording electrode and Lee et al. (2017) employing an 8 × 8 multi-electrode array, which was positioned under the hippocampal slice. The latter arrangement would probably have resulted in greater variation in recording sites across CA1. The time points were different, with Zheng et al. (2003) using 4–6 weeks or 5–6 months following the lesion and Lee et al. (2017) using 1 day, 1 week, and 1-month post-lesion. Despite the methodological differences, the two studies showed similar changes in excitability in the hippocampus, albeit that LTP induction was not specifically measured in Zheng et al. (2003).

### *In vivo* Studies

Unlike the *in vitro* studies, the *in vivo* studies have mainly concerned LTP in the hippocampus following BVL rather than UVL (Zheng et al., 2010; Truchet et al., 2019). Notably, neither of the *in vivo* studies found the decrease in fEPSP slope or PS amplitude without LTP, that was reported in the *in vitro* studies (Zheng et al., 2003; Lee et al., 2017), which is very interesting. This suggests that UVL and BVL may have completely different effects on the intrinsic excitability of the neurons in the hippocampus, perhaps due to the asymmetrical effects of UVL on the CNS.

The results of the two *in vivo* studies of LTP following BVL are also inconsistent with each other. Zheng et al. (2010) observed no effect of a complete BVL on LTP in the DG or CA1, whereas Truchet et al. (2019) observed an increased PS relative to the fEPSP following LTP in the DG. However, there is a multitude of methodological differences between these two studies. First, while Zheng et al. (2010) employed a surgical BVL, Truchet et al. (2019) used a chemical BVL. A major difference between the surgical and chemical BVLs used in these two studies is the timing between the lesions to the two sides of the vestibule. The surgical BVL lesioned both sides during one surgery with one inner ear lesioned about 30 min before the other, while the chemical BVL was performed on one side before the second side was lesioned several weeks later. Therefore, the animals would experience UVL on one side first and undergo vestibular compensation before experiencing a 2nd UVL and vestibular compensation on the other side. Given the fact that the two UVL studies (Zheng et al., 2003; Lee et al., 2017) both reported changes in neuronal excitability in the hippocampus, while the BVL studies did not (Zheng et al., 2010; Truchet et al., 2019), it is perhaps not surprising that there might be a difference in LTP induction between surgical and chemical BVL animals. Also, surgical BVL involves the surgical destruction of the vestibular system, while trying to avoid damage to the cochlea, whereas the injection of sodium arsanilate or any other ototoxin through the tympanic membrane is less well controlled and incurs unknown damage to the cochlea. Second, Zheng et al. (2010) measured LTP for 30–60 mins at multiple time points post-UVL, including up to 43 days in alert, freely moving animals and then at 7 months in anesthetized animals, whereas Truchet et al. (2019) measured LTP for 3 hs at 1 time-point (3 months) in anesthetized animals following BVL. Third, whereas Zheng et al. (2010) quantified LTP in both CA1 and the DG, Truchet et al. (2019) quantified it only in the DG. Fourth, the two studies used different LTP induction protocols. While the inter- and intra-train frequencies and the pulse durations were the same in both studies, Zheng et al. (2010) used twice as many HFS trains compared to Truchet et al. (2019); 20 vs. 10, for a respective total of 200 and 100 pulses. Therefore, it can be argued that Zheng et al. (2010) used a high-frequency protocol intended to generate a strong LTP, reflected in the higher level of LTP obtained for both the fEPSP and PS in their study. By contrast, Truchet et al. (2019) attempted to induce a “weak” or “moderate” LTP in order to avoid a possible ceiling effect. The fact that Truchet et al. (2019) observed an enhanced LTP of the PS suggests that such an effect might be missed by a ceiling effect in the Zheng et al. (2010) study due to the stronger LTP induction paradigm used.

Tai and Leung (2012) found that natural vestibular stimulation could enhance LTP in the hippocampus *in vivo*. This effect appeared to be mediated by cholinergic input from the medial septum, since an ACh receptor antagonist blocked the effect, as did selective lesions of the septum. How this result might relate to those of Zheng et al. (2010) and Truchet et al. (2019) is unclear. If the results obtained by Truchet et al. (2019) are interpreted as dysfunctional LTP due to reduced input selectivity and increased synaptic noise, then the Tai and Leung (2012) finding that vestibular stimulation increases LTP, could be interpreted as the opposite effect. However, the data obtained from these two studies and those of Zheng et al. (2010) are not necessarily incompatible: when it comes to LTP and memory, the relevant question is not only whether there is more or less LTP, but rather which synapses express LTP. Indeed, a general increase in synaptic efficacy (reflected by a global increase in LTP), due to NMDA receptor upregulation following BVL, can lead to an increase in synaptic noise and therefore learning and memory deficits. On the contrary, natural vestibular stimulation could generate plasticity more similar to that which develops during natural vestibular-hippocampal interactions, specific to synapses involved in learning and memory processes. One must also remember that LTP is generated from an artificial stimulation protocol and that minor variations could result in different effects on LTP.

## NMDA Receptors in The Hippocampus Following BVL

Like the studies of LTP, there are only a few studies of hippocampal NMDA receptors following a vestibular loss. These can only be considered correlational concerning LTP since they were conducted in animals not subjected to LTP-inducing stimuli.

Although some early studies examined NMDA receptor subunit density in the hippocampus at time points up to 2 weeks post-UVL (Liu et al., 2003), the major studies have used BVL. The latter studies have focussed on longer time points due to the interest in long-term spatial memory deficits caused by vestibular loss (for reviews see Besnard et al., 2016; Smith, 2017; Agrawal et al., 2020), and these studies will be emphasized here. Besnard et al. (2012), using receptor autoradiography, observed an increase in the B_max_ for NMDA receptors in the hippocampus at 2 months following BVL, which involved two sequential UVLs. Benoit et al. (2018a) and Truchet et al. (2019) recently reported similar results, using either flow cytometry or receptor autoradiography, or both, at 7 and 30 days following BVL. By contrast, Zheng et al. (2013) could find no difference in any NMDA receptor subunit in any subregion of the hippocampus at 24 h, 72 h, 1 week, 1 month, or 6 months following BVL. Although the different studies involve methodological differences such as chemical (Besnard et al., 2012; Benoit et al., 2018a; Truchet et al., 2019) vs. surgical lesions (Zheng et al., 2013) of the vestibular system as well as different time points post-BVL, the most important difference is probably the use of receptor autoradiography (Besnard et al., 2012; Truchet et al., 2019) and flow cytometry (Benoit et al., 2018a,b; Truchet et al., 2019) vs. western blotting (Zheng et al., 2013). While western blotting permits the use of antibodies to target specific NMDA receptor subunits (as does flow cytometry), it involves quantification of the total amount of protein in the homogenate, including that which is not specific to synapses. By contrast, receptor autoradiography targets receptors that are more likely to be synaptic and therefore may be more representative of NMDA receptors involved in LTP, although this is not certain. On the other hand, only western blotting and flow cytometry can provide information about NMDA receptor subunits. In the end, none of these studies can be related to LTP directly, since they were conducted in animals in which electrophysiological recording was not performed.

## Conclusions

It is difficult to draw firm conclusions from the few studies of hippocampal LTP following UVL or BVL, conducted so far. Although UVL appears to cause a decrease in hippocampal field potentials following UVL in *in vitro* hippocampal slices (Zheng et al., 2003; Lee et al., 2017), this has not been demonstrated following BVL *in vivo* (Zheng et al., 2010; Truchet et al., 2019) and no UVL *in vivo* investigation has been conducted. The study by Zheng et al. (2010) involved both CA1 and the DG, so the discrepancy is unlikely to be due to the subregion of the hippocampus studied. More likely, the difference may be due to the use of UVL vs. BVL. As mentioned earlier, UVL causes a massive asymmetry between the two VN in the brainstem, which is then transmitted to higher brain regions, whereas BVL removes all vestibular input simultaneously but does not cause such an asymmetry.

Despite the plethora of methodological differences among the LTP studies, the major discrepancy between the two *in vivo* studies is most likely the different lesioning methods and HFS protocols used to induce LTP. It may be that differences in LTP can be detected, depending on the stimulation protocol used. If an increase in ES potentiation does occur following LTP, at least using a “moderate” HFS protocol, then it may be related to a decrease in feed-forward inhibition mediated by GABAergic interneurons acting on GABA_A_ receptors on principal cells (Tomasulo et al., 1991; Lu et al., 2000; Ross and Soltesz, 2001). This could happen if BVL caused a reduction in these GABA_A_ receptors. Previous studies of the CA1 following BVL have shown a significant increase in the spontaneous resting activity of interneurons (Russell et al., 2003), which also suggests that interneuron regulation of principal neurons may change. DG and CA1 hippocampal subfield circuitries display feedforward and feedback inhibition (GABAergic interneurons) of their main cell types, respectively granule and pyramidal cells. Perforant path axons directly connect with inhibitory interneurons underlying the feedforward inhibition. These interneurons fire with very short latencies and their action could control the granule cell activity. Moreover, blocking GABA_A_ receptors in the DG *in vivo* prevents the leftward shift of the ES curves during LTP induction (Tomasulo et al., 1991). The authors proposed “…*that the LTP-associated left shift of the ES curve reflects a real decrease of synaptic efficacy in the inhibitory pathway*” (Tomasulo et al., 1991). Lu et al. (2000) showed that calcineurin activated by NMDA receptors induced long-term depression (LTD) of the GABA_A_ receptor-dependent inhibitory post-synaptic potential (IPSP) during NMDA receptor-dependent LTP in CA1, and stated that “…*this LTD is both necessary and sufficient for the long-lasting increase in enhanced excitability manifest in the E-S coupling*” (Lu et al., 2000).

The results of the studies of NMDA receptor expression in the hippocampus are inconsistent. If there is an increase in synaptic NMDA receptors following BVL, this may cause an increase in synaptic noise that would lead to spatial memory deficits. Future studies should further investigate the effects of UVL and BVL, on LTP, especially *in vivo*, in the different hippocampal subregions and changes in feed-forward GABAergic inhibition. It would be particularly interesting to investigate the effects of restoring GABAergic inhibition in the hippocampus following UVL or BVL, using selective agonists or optogenetics, on E-S potentiation and synaptic noise (Truchet et al., 2019).

## Author Contributions

PS conceived and wrote the first draft of the article. BT, FC, YZ, and SB contributed to the writing and editing of the manuscript.

## Conflict of Interest

The authors declare that the research was conducted in the absence of any commercial or financial relationships that could be construed as a potential conflict of interest.
